# Catching and throwing exercises to improve reactive balance: A randomized controlled trial protocol for the comparison of aquatic and dry-land exercise environments

**DOI:** 10.1371/journal.pone.0275733

**Published:** 2022-10-12

**Authors:** Youngwook Kim, David A. E. Bolton, Michael N. Vakula, Eadric Bressel

**Affiliations:** Department of Kinesiology and Health Science, Utah State University, Logan, Utah, United States of America; Public Library of Science, UNITED KINGDOM

## Abstract

Reactive balance, a critical automatic movement pattern in response to a perturbation, is directly linked to fall prevention in older adults. Various exercise interventions have been broadly performed to improve reactive balance and thus prevent falls. Curiously, aquatic exercises have been suggested as an effective balance intervention and a safer alternative to exercises on dry land yet the efficacy of aquatic exercises on reactive balance has not been formally investigated. The present clinical trial aims to identify if skills acquired during aquatic exercise are more effectively transferred to a reactive balance task than land exercise. This study is designed as a double-blinded, randomized controlled clinical trial. Forty-four older adults aged 65 years or above who meet the eligibility criteria will be recruited and randomized into an aquatic exercise group or land exercise group. Each group will participate in the same single bout intervention that includes a ball throwing and catching task. A modified lean-and-release test will be implemented on land immediately before, after, and one week after the single bout intervention. The outcomes will include reaction time, rapid response accuracy, and mini-BESTest scores obtained from stepping and grasping reactions. All statistical analyses will be conducted using an intention-to-treat approach. Our conceptual hypothesis is that participants in the aquatic exercise group will demonstrate more improved outcome scores in the lean-and-release test when compared to those in the land exercise group. The results of the present study are expected to provide evidence to support the benefits of aquatic exercises for improving reactive balance in older adults. Further, participants may find aquatic exercises safer and more motivating, thus encouraging them to participate in further aquatic exercise programs.

## Introduction

### Background

In daily life, reactive balance control strategies are required in response to a variety of postural perturbations, such as slips, trips, or bumps (pushes or pulls), to avoid potential falls while sitting, standing, or walking. According to WHO Global Report on Falls Prevention in Older Age, risk factors associated with physical environments (e.g., slippery surfaces, unmarked obstacles, and uneven sidewalks) play the most significant role in falls in older adults [1]. Reactive balance, which can be defined as an ability to recover a stable postural balance in response to a mechanical perturbation, requires the rapid generation of postural muscles in the trunk, lower, or upper limbs to induce proper reactions, such as swaying around the ankle or hip joints, taking a reactive step, or reaching to grasp a handhold [2]. Delayed and inappropriate generation of corrective forces from the postural muscles may result in falling and injuries [3]. Reactive balance control is supported by visual, vestibular, or somatosensory feedback, and it can be improved by repeated exposure to perturbations not only in healthy young and older adults, but individuals with neurological diseases, such as multiple sclerosis [4].

### Ball throwing and catching exercise

In various clinical settings, exercises using ball throwing and catching activities have been broadly used to improve balance, gait, or proprioceptive functions and to reduce the risk of athletic injuries [5,6]. However, the number of balance exercise studies that utilize ball throwing and catching activities is limited, and there is still a need for further investigation of the effects of the activities on various balance outcomes. Ball throwing and catching exercise engages two different motor tasks, including targeted grasping reactions as a primary motor task and postural shifts as a secondary motor task, in conjunction with cognitive demands regarding perceptual processing of the direction of the ball projection. Activities of daily living generally necessitate balance control during one or more different tasks, thus, it has been identified that deteriorated balance control during dual-task testing is associated with future fall risk in older adults [7]. Several researchers have recently conducted balance exercise studies in healthy young adults [8], healthy older adults [9,10], older adults with mild cognitive impairment [11], stroke patients [12], and multiple sclerosis patients [13] using self-initiated or external postural perturbations to improve reactive balance, described as compensatory postural adjustments. They utilized a ball throwing activity as a self-initiated, internal postural perturbation and a ball catching activity as an external perturbation. The ball throwing and catching exercises improved both anticipatory and compensatory postural adjustments in the aforementioned populations, which was demonstrated by early onsets of postural (i.e., leg and trunk) muscle activities, larger anticipatory center of pressure (COP) displacements, and smaller compensatory COP and center of mass (COM) displacements. However, the participants, specifically the older adults or those with neurological diseases, in the studies were exposed to potential risks, such as losing balance and falling during the intervention and testing sessions conducted in a dry-land environment.

### Aquatic exercise

Exercises performed in an aquatic environment have been suggested as an effective alternative to exercises on dry land for people with a variety of clinical conditions as it offers a safer environment if one were to fall [14,15]. The effectiveness of aquatic exercise (AE) is fundamentally attributed to the physical properties of the water [14]. For example, the buoyancy of the water provides a supportive force and a reduction in joint loading. Viscous force and hydrostatic pressure enhance sensory and proprioceptive awareness, which may be beneficial to the postural control system [16]. In addition, the aquatic environment slows movements and enhances participants’ confidence level (i.e., decrease in fear of falling) by potentializing their ability to attempt movement outside their base of support [16]. Given these advantages of the aquatic environment, aquatic exercises have been widely implemented in the early stages of rehabilitation or used as a paradigm of aquatic cross-training [16,17]. Further, aquatic exercises are specifically advisable for those who cannot tolerate land-based exercises or strive for novel training stimuli.

The dynamic systems theory provides some additional justification to examine if an aquatic environment may confer some advantages over the land environment for improving reactive balance in response to a ball throwing and catching exercise. According to the dynamic systems theory, variability in the movement systems affects each individual’s behavior and consequently helps one adapt to ever-changing constraints (personal, task-related, or environmental) [18]. For example, gait training with a specific perturbation carries over to post-perturbation performance during walking [19]. The aquatic environment appears to increase the variability in movement behavior and therefore requires our neuromuscular system to adapt to it [20–23]. In general, less-skilled performers (e.g., older adults) or individuals with a certain disease, illness, or injury demonstrate diminished ability in utilizing appropriate biomechanical degree of freedom and consequentially weakened adaptability to task constraints [18]. Thus, for these populations, exercise interventions in an aquatic environment may be more effective at improving post-intervention performances regarding reactive balance in various circumstances versus those in a dry-land environment. Despite the abovementioned attributes and benefits of the aquatic environment, there is a void in the literature regarding how effectively aquatic exercises can be transferred to different tasks in a dry-land context when compared to land exercises.

### Problem statement

Specificity of training is a key element in improving balance, and it is particularly emphasized in perturbation-based reactive balance exercise interventions [24]. Thus, results from the abovementioned studies utilizing ball throws and catches as postural perturbations are noteworthy in that all of the results showed improvements in similar tasks [8–13], and three of which additionally demonstrated the transfer of the learning effects of the exercise interventions to another task, that was not a part of the interventions, but sharing the same directional specificity [9,10,13]. The number of perturbations and contexts that can be furnished during exercises is limited in conventional clinical or research settings. Hence, there is a need for further research to investigate the effectiveness of exercises that are broadly implemented in various clinical settings without environmental constraints on reactive balance.

In this study, reactive balance will be assessed using a modified lean-and-release test [25]. During the test, there are two options that participants can take, including reactive stepping and reach-to-grasp reaction, to avoid a fall. Moreover, cognitive processes are required to override a prepotent action (i.e., response inhibition) and select a suitable response (i.e., action selection) considering the available environmental affordances and constraints. A ball throwing and catching exercise in the current study will introduce these aforementioned components. While the previous studies included fixed-support reactive balance exercises and assessments, the single bout intervention in this study will require both fixed-support and change-in-support strategies. Both the test and exercise require eye-foot coordination (i.e., stepping) and/or eye-hand coordination (i.e., reach-to-grasp and ball catch) based on the environmental affordances, and participants will be required to maintain balance to avoid a fall after each reaction. Additional cognitive processes will be demanded via deceptive actions. The directions of the ball will be random, and some trials will include a deceptive action before the actual throw thus forcing a need for rapid response inhibition and action selection when they shift and catch the ball. During the single bout intervention, both the speed and accuracy of the movement will be emphasized.

Hence, the current study aims to compare measures of reactive balance control after a single bout intervention on land and in chest-deep water. There have been several studies presenting greater enhancements in dry-land balance performance, specifically in static and proactive balance measures, such as Berg Balance Scale [26], 8-Foot Up and Go Test [27], and Functional Reach Test [28] following AE versus land exercise (LE) in healthy older adults. As none of the previous trials included any measures of reactive balance, the current study will specifically focus on improvements in reactive balance capacity after a single bout intervention. During aquatic exercises, water resistance produces overload during movements, and hydrostatic pressure constantly facilitates sensory awareness and motor functions. Further, the confidence level is augmented in the safer aquatic environment, which in turn increases variability in movement systems and maximizes neuromuscular adaptations. Thus, we hypothesize that participants in an AE group will present greater improvements (e.g., more accurate and rapid responses) during the reactive balance assessment compared to those in a LE group. The greater improvements will be demonstrated by faster response speed (i.e., shorter reaction time), greater accuracy alongside faster response speed (e.g., higher rapid response accuracy), and greater quality of response (e.g., higher mini-BESTest score). The Mini Balance Evaluation System Test (mini-BESTest) is a comprehensive clinical balance assessment, which evaluates four different balance control systems. This tool is commonly used by clinicians and practitioners and well known to be advisable to identify the disordered systems underlying balance control and design specific rehabilitation approaches in varied age and patient populations [29]. In this study, the second section (Reactive Postural Control) will be utilized to score the quality of compensatory balance responses.

## Materials and methods

### Study design and setting

This study will be a double-blinded randomized controlled trial, implemented in the Sorenson Legacy Foundation Center for Clinical Excellence at Utah State University (Logan, Utah, United States). The protocol was designed in accordance with Standard Protocol Items: Recommendations for Interventional Trials (SPIRIT) statement (S1 Appendix) [30] and will be reported according to the Consolidated Standards of Reporting Trials (CONSORT) recommendations [31]. The trial results will be disseminated at national and/or international academic conferences and in peer-reviewed journals.

### Participants

A total of 44 individuals aged 65 years or above will be asked to participate in this study. Participants will be recruited from university and community settings via written flyers or word-of-mouth referrals. When a participant expresses interest in the study by contacting one of the researchers via the email address listed on the flyer, we will send a copy of the consent form via email to allow the prospective participant the opportunity to read the consent form and become fully informed before participating in the study. The estimated amount of time between first receiving a copy of the consent form and signing it in person at their appointment will be approximately one week.

Participants will be included in the study if they: 1) have the ability to stand using a double-leg stance for one minute of time, 2) walk independently, 3) have normal or corrected to normal vision, and 4) normal or corrected to normal hearing based on a qualitative assessment. Exclusion criteria will include: 1) any neurological or musculoskeletal disorders that may inhibit the participation in the single bout intervention and testing protocols; 2) a concussion within the past one year before the participation; 3) any cognitive deficiencies (e.g., memory, concentration, or attention disorder); 4) one or more ‘yes’ answered on the Physical Activity Readiness Questionnaire (PAR-Q); or 5) fear of water. To fully assess each participant’s eligibility, a pre-screening questionnaire will be additionally completed.

### Sample size calculations

A power analysis was conducted using G*Power software package (Version 3.1.9.7, Kiel University, Germany). Using results from previous work [26], an a-priori power calculation (α = 0.05, and 1-β = 0.80) determined that 38 participants (19 per group) will be required to determine significant intergroup differences based on large effect size (Cohen’s d > 0.8). Berg Balance Scale (BBS) score data from an AE and LE group were extracted for the analysis as (1) there was no study comparing the effects of AE and LE on any measures of reactive balance and (2) BBS is commonly considered to be the gold standard in testing functional balance and measuring fall risk in older adults [32]. Considering an approximately 15% dropout rate, a total of 44 participants (22 in each group) will be expected to enter the study.

### Ethical approval and registration

The procedures described in this protocol conform to the principles outlined in the Declaration of Helsinki and received approval from the Institutional Review Board at Utah State University (IRB: 12227). All participants will read and sign a written informed consent approved by the Institutional Review Board at Utah State University before participating in the experimental procedures. The protocol was registered on the U.S. National Library of Medicine (ClinicalTrials.gov) with the identifier (NCT05107817) on 3 November 2021. The individual in this manuscript has given written informed consent (as outlined in PLOS consent form) to publish these case details.

### Timeline

**Fig 1:**
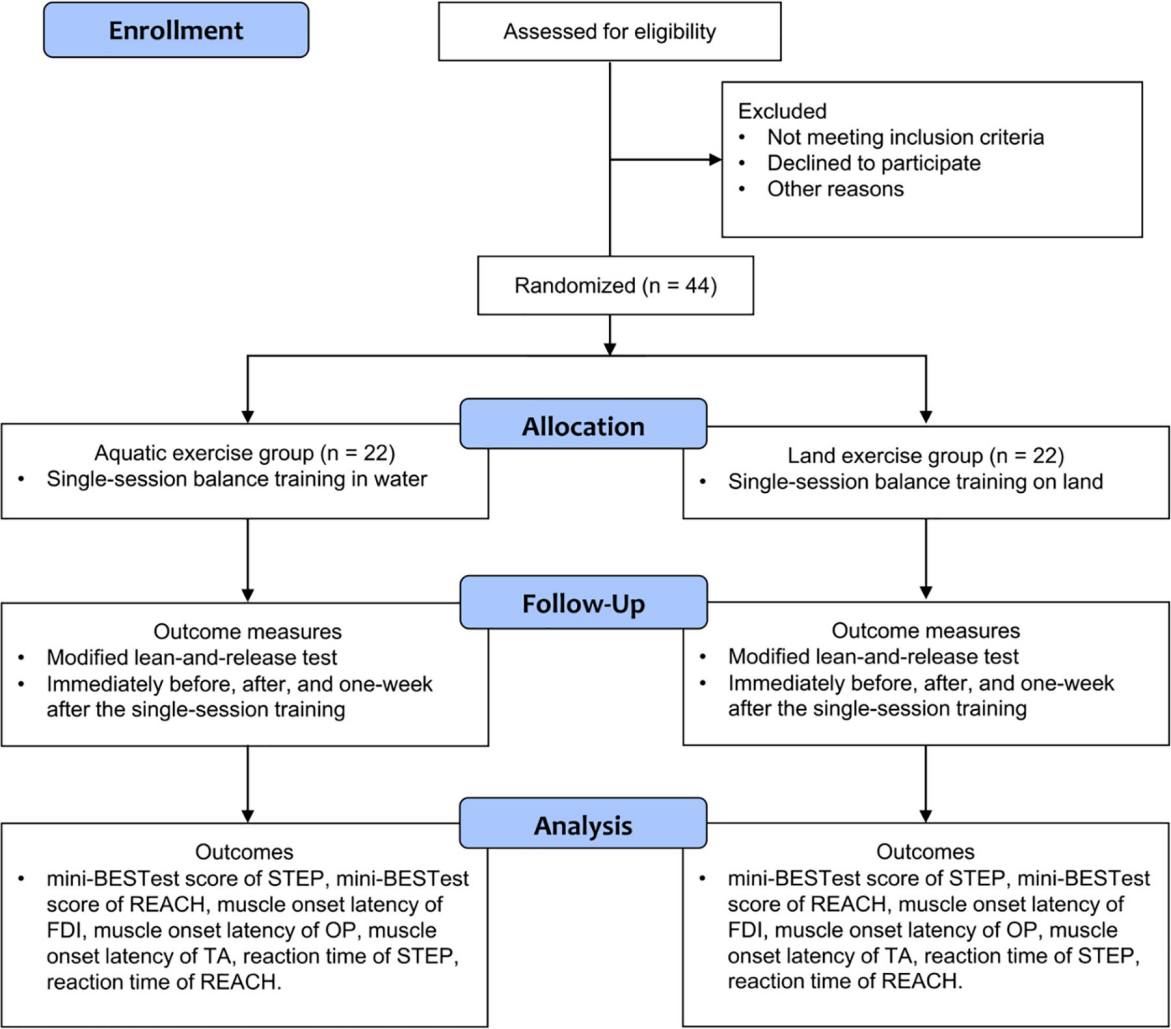
CONSORT flow diagram of the planned protocol timeline.

The process of enrollment, allocation, follow-up, and analysis are outlined in Fig 1. The participant recruitment is expected to continue from December 2021 to December 2022.

### Randomization and allocation concealment

An experimenter, a certified athletic trainer, will screen according to the aforementioned eligibility criteria using a pre-screening medical history questionnaire and PAR-Q. Following the baseline screening, all participants meeting the eligibility criteria will be randomly assigned to either AE or LE group. Simple randomization will be applied using Random Allocation Software [33]. The numbers associated with the allocation sequence and identities will be kept in sealed envelopes and concealed until completion. Randomization and allocation will be implemented by an independent researcher not involved in any part of the study.

### Blinding

Participants and an assessor who will collect the outcome measures at the baseline, post-intervention, and follow-up and analyze the data will be blinded to the allocation. It will be impossible to fully blind the exercise instructors due to the nature of this study and their active role in the single bout intervention.

### Intervention

The AE will be executed in a climate-controlled aquatic research laboratory, and the LE will be performed in a motion analysis laboratory. During AE, participants will stand barefoot on an adjustable floor of an aquatic treadmill (HydroWorx 2000TM, Middletown, PA), and the water depth will be adjusted to each participant’s xiphoid process level. Participants will sit on a chair for five minutes before AE for environmental acclimatization.

Participants will engage in a single bout intervention consisting of 120 repetitions of a ball throwing and catching task using a volleyball (Wilson Sporting Goods Co., Chicago, IL, USA). This single-session approach was selected based on a previous study, reporting the long-lasting effects of a single bout intervention with an intense reactive balance exercise [34]. Several previous studies also demonstrated that reactive balance in response to a postural perturbation has been improved after a single bout intervention [8,9,11–13].

The participants will be asked to throw the ball at a self-paced high speed at the chest-to-shoulder level using two hands towards the trainer’s hands positioned at a 3-meter distance from them and 20-degree above an imaginary horizontal line at each participant’s eye level. For the consistent height and trajectory of the ball across all sessions, the trainer will kneel on the floor in front of the pool during AE sessions and stand on a step box during LE sessions. Then, they will catch the ball thrown at a moderate speed by the trainer directly toward the midline of their body, to the left or right of the midline, or to the closer or farther point from the trainer to induce fixed-support reactions and change-in-support reactions in both frontal and sagittal planes of the participants. The directions of the ball will be random and unpredictable, and the number of the directions will be proportionately included. After each repetition involving one throw and one catch, participants will be asked to return to the start position for the next repetition. The intervention session will be composed of 3 sets of 40 repetitions with a 2-min break after the first and second set. Of the 120 repetitions, the catches will follow 60 direct throws and 60 deceptive throws from the trainer to introduce the concepts of response inhibition and action selection. For example, a deceptive throw will involve a deceptive action that provides misleading information about the direction of the ball projection (e.g., a throwing motion to the right without an actual throw) before the actual throw (e.g., throwing the ball to the left, which is the predetermined direction). While catching the ball, the participants will be allowed to select whichever strategy they wish and freely move their bodies to properly react and catch the ball. The Borg Scale for Rating of Perceived Exertion (RPE) will be used during each intervention set, and the heart rate will be recorded using a finger pulse oximeter (SportStat; Nonin Medical, Inc., Minneapolis, MN. USA) before and after each set to subjectively and objectively monitor the intensity of the exercise. Additional rest will be given if needed. The exercise intervention procedures used previously in several trials have been reproduced and modified to generate reliable results in accordance with our research purpose [9,11–13]. After each intervention session, subjective measures of satisfaction, difficulty, and safety levels will be additionally performed using a visual analogue scale (VAS, range: 1–10).

### Outcome measurement

**Fig 2:**
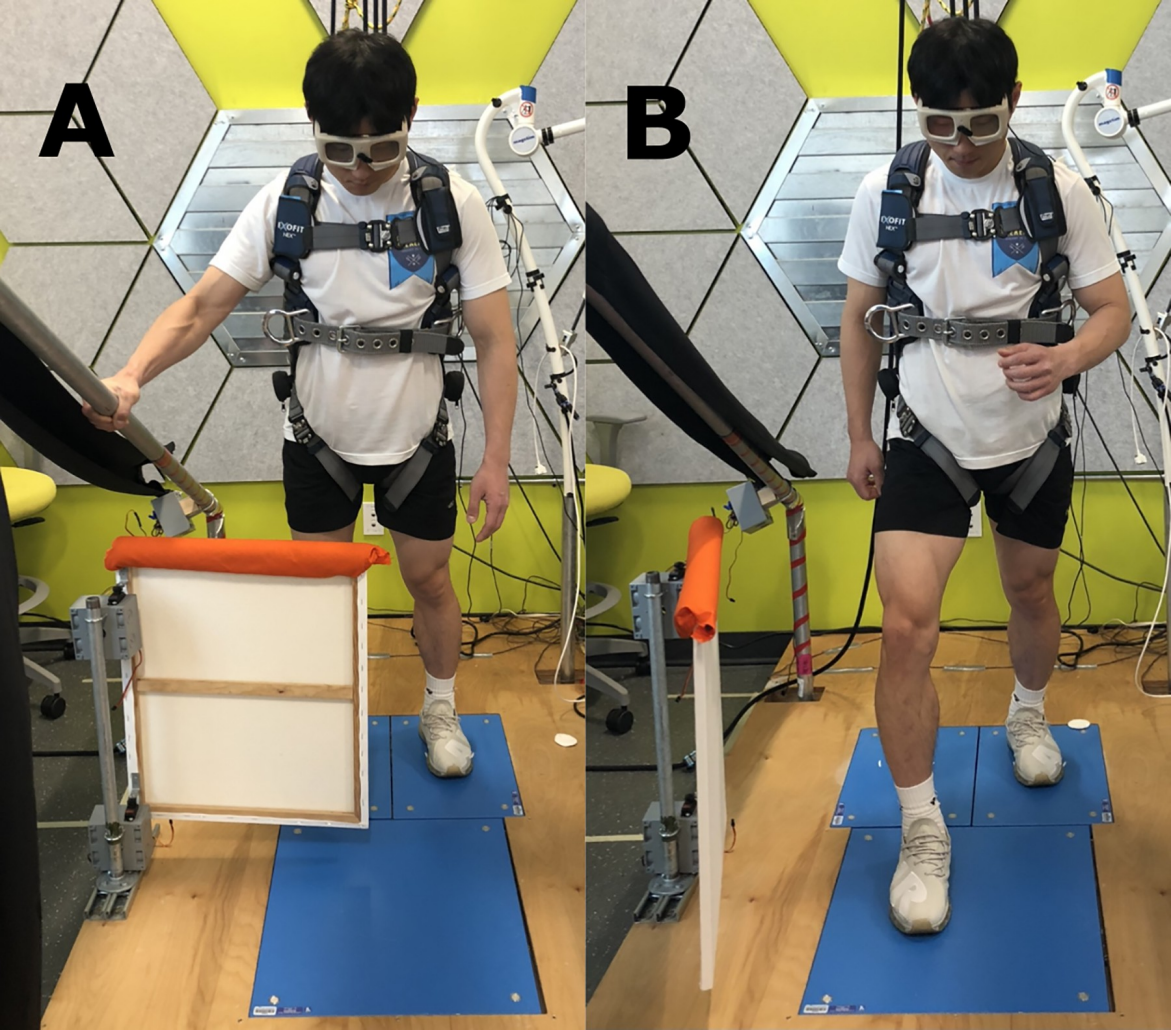
Modified lean-and-release task. (A) Grasping a safety handle using right hand while maintaining both feet fixed. (B) Stepping forward using a preferred leg.

A modified lean-and-release technique devised by Bolton and Mansour (2020) will be implemented on land to assess reactive balance immediately before (pre), after (post), and one week after (follow-up) the single bout intervention [25]. Lean-and-release techniques are commonly used to assess reactive balance in various older adult populations [35–37]. This modified lean-and-release technique additionally introduces cognitive factors (i.e., response inhibition and action selection), which are important in certain situations. For safety purposes, participants will wear a harness, which is secured to the ceiling to prevent them from falling in case they fail to recover balance. The participants will then stand with both feet hip-width apart on two separate, parallel force plates (type 9260AA, Kistler AG, Winterthur, Switzerland). A horizontal cable will be attached to the safety harness at the midthoracic level and the other end to the wall behind the participants by a magnet. To simulate a forward fall (trip) situation, the experimenter will instruct participants to lean forward into a support cable while keeping both feet in contact with the floor and to remain relaxed (Fig 2). This position will require dorsiflexion of the ankle, as the rest of the body remains aligned. The forward lean position for each participant, measured by the ankle joint angle, will be determined as the minimal lean angle where a change-of-support reaction (i.e., forward step) is necessary to recover balance upon cable release [38]. Then, the magnet will be deactivated to suddenly release the cable. There are two possible settings: 1) the leg block is placed in front of both legs, and a safety handle is uncovered; or 2) the leg block is removed, and the safety handle is covered. The leg block and handle cover will be controlled via computer-triggered, servo motors. The testing session will be comprised of three blocks: 1) REACH (grasping a safety handle using their right hand while maintaining both feet fixed), 2) STEP (stepping forward using any leg), and 3) RANDOM (random variations of STEP and REACH). To control vision during the third block, participants will wear liquid crystal goggles (Translucent Technologies Inc. Toronto, ON, Canada), that will be closed at the beginning of each trial and opened 400 ms before the cable is released. An experimenter will make careful note of the stepping leg used on each trial, and the participants will be free to step with either leg during testing. The timing of foot-off of the stepping foot will be recorded using the two parallel force plates. A force-sensitive resistor (B&L Engineering, Santa Ana, CA, USA) placed on the top surface of the safety handle and one large force plate in front of the two parallel force plates will be used to detect hand contact with the safety handle during REACH and foot contact during STEP, respectively. To control the predictability of the forthcoming reactions during the third block, the settings will be randomly changed between trials via the data collection program. After sufficient practice trials to familiarize the participants with the stepping and reaching actions, five trials each of the REACH and STEP will be completed, and ten actual trials of RANDOM, including five STEP and five REACH actions in random orders, will be completed. Outcome measurements at post and follow-up will include all randomized participants within the original groups regardless of any discontinuation or deviation from intervention protocols.

During the testing, the quality of the compensatory reactions will be scored using the Reactive Postural Control section of the mini‐BESTest [39,40]. The scores will be ranged from 0 to 2. The scoring criteria for the STEP task will be as follows: 0) no reactive step or falling; 1) more than one reactive step; and 2) one successful reactive step. For the REACH task, the criteria were modified as follows: 0) no grasp reaction or falling; 1) grasp reaction with one or more reactive steps; 2) successful grasp reaction without a reactive step. During the RANDOM block, the number of accurate responses will be recorded. The testing will be video-recorded and assessed by two experimenters, who are blinded to the allocation.

### Data processing and analysis

Force data in relation to the cable release will be extracted for further analysis to define the reaction time. Foot-off and foot contact will be defined as the time (ms) following the cable release at which the force under the stepping foot becomes less than and greater than 1% of the body weight, respectively [40]. Hand contact with the handle during REACH will be defined as the time following the cable release at which the force sensitive resistor detects a force.

For the RANDOM block, response accuracy, defined as the percentage of accurate responses, will be additionally calculated. To represent a composite measure of accuracy and speed of response, rapid response accuracy will be calculated using the ratio of response accuracy to the reaction time (%/ms), similar to the approach recently developed in a device measuring hand reactions (46). Foot-off and hand contact data will be used for the calculation.

### Data management and confidentiality

All data will be collected using computer-based data acquisition programs. This data will be securely stored in a restricted-access folder on Box.com, an encrypted, cloud-based storage system. Any physical content (e.g., pre-screening questionnaires and informed consent documents) will be stored in a locked drawer in a restricted-access office. These documents will not be shown to any individual other than the investigators or the participants. Each participant will be assigned a participant number and all data files will be named according to the number such that the identity of the participants will only be apparent to observers other than the investigators. Important protocol modifications prior to the completion will be communicated with the trial registry and the journal of publication. The confidentiality of the personal information will be protected by the principal investigator. All investigators will have access to the final dataset. A data monitoring committee is not needed as minimal risk applies to this protocol.

### Safety assessment

To minimize any risk of falls, a spotter will be positioned behind the participants and cushion mats will be positioned around them during the land exercise session. There is no risk of falls during the aquatic exercise session. However, a spotter, who is certified in CPR, Basic Life Support, or Lifeguard, will be positioned behind the participants during the aquatic exercise session. During the testing sessions, participants will wear a harness, which is secured to the ceiling to prevent them from falling in case they fail to recover balance. Any adverse events or unintended effects will be monitored throughout the study, follow-up, and four weeks after the intervention and discussed in the final registered report research article.

### Statistical analysis

Dependent variables included in the statistical analyses will be: 1) hand contact time during REACH; 2) foot-off time during STEP; 3) foot contact time during STEP; 4) rapid response accuracy during RANDOM; 5) mini-BESTest score of REACH; 6) mini-BESTest score of STEP; 7) mini-BESTest score of RADOM. There are two independent variables including the groups (AE vs. LE) and time (pre vs. post vs. follow-up).

The baseline differences in demographic data and all dependent variables between AE and LE groups will be analyzed using independent samples t-tests for data with a normal distribution and using the non-parametric Mann-Whitney U-test for data without normal distribution. On confirming the assumptions of the normal distribution (Kolmogorov–Smirnov test) and homogeneity of variances (Levene’s test), dependent variables will be compared using 2 (group: AE, LE) × 3 (time: pre, post, follow-up) repeated-measures (RA) analysis of variance (ANOVA) with group as a between-group factor and time as a within-group factor. If baseline differences of the dependent variables occur, baseline values will be included as covariates. If the assumptions are violated, Kruskal-Wallis one-way ANOVA will be used for nonparametric variables. In addition, post-hoc analyses will be conducted using Tukey post hoc test or Mann-Whitney U test. The meaningfulness of statistical differences will be reported using Cohen’s d effect sizes (ES) and confidence intervals (95%) for the parametrical variables and probability of superiority for dependent samples (PS_dep_) for the nonparametric variables [41]. Alpha will be set *a priori* at .05 for all statistical analyses. Missing data will be handled using multiple imputation, and all statistical analyses taking an intention-to-treat approach will be conducted with SPSS version 25 (SPSS Inc., Chicago, IL, USA).

## Discussion

To the best of our knowledge, this will be the first study to compare the transferability of aquatic-based and land-based reactive balance exercises on a different reactive balance task in older adults. More specifically, this study will quantify the effectiveness of a single bout intervention in water and on land on reactive balance performances, closely associated with fall prevention strategies in real-life settings, and how much it can be retained after termination of the training.

Although multifactorial exercise is the recommended form of intervention for preventing falls in older adults [42], reactive balance in specific circumstances cannot be pretermitted in exercise-based interventions. When the body loses balance, reactive balance, which is mostly exhibited in the form of reactive stepping or reach-to-grasp action, is the last line of defense to prevent a fall regardless of the cause of falls [24]. However, motor skills trained in a specific context can be transferred to an untrained task only to a limited degree. Considering the variety of mechanical postural perturbations in real-life contexts, in regards to the direction, magnitude, or point of application of the force, further studies are required to potentially corroborate and expand our results to different types of postural perturbations that commonly cause falls in older adults’ daily life.

Older adults, without regard to any clinical conditions, are always exposed to risks of falls, that are attributable to age-related neuromuscular degenerations. Thus, given the potential fall risk factors especially related to physical environments during reactive balance exercises in a dry-land condition, understanding and developing effective exercise-based interventions in a safer environment (i.e., aquatic environment) is paramount to engaging older adults in advanced balance training and ultimately prevent falls and improve their quality of life. Recently discovered evidence from a meta-analysis has suggested that aquatic exercises may be used as an effective, safer alternative to conventional land-based exercises in the elderly [15]. The results of this study may have implications for exercise prescriptions aimed at aging individuals who aspire to participate in physical activities to improve balance and prevent falls but have fear of falling or kinesiophobia.

## Supporting information

S1 Appendix SPIRIT checklist. (DOC)

S1 File (PDF)
