# Supplementary material for: Catching and throwing exercises to improve reactive balance: A randomized controlled trial protocol for the comparison of aquatic and dry-land exercise environments
Source: PLoS One. 2022 Oct 12;17(10):e0275733. doi: 10.1371/journal.pone.0275733 (PMC9555657; doi:10.1371/journal.pone.0275733)
Supplement: S1 File — (PDF) [file pone.0275733.s002.pdf]

EFFECTS OF AQUATIC EXERCISE ON BALANCE CONTROL

Introduction

The apparent ease at which many people perform activities of daily living would suggest the internal and external environments in which these activities are performed are favorable for successful human movement. To the contrary, when a person stands or performs bipedal gait (e.g., walking), they use multiple joint segments, a small base of support, and a relatively high center of gravity, and they are confronted with many destabilizing forces (perturbations) from the environment (e.g., uneven surfaces) or movements (e.g., throwing or kicking motions). Neuromechanically speaking, this scenario presents a maximal threat to balance control, and unsurprisingly, researchers have observed that balance control is associated with a myriad of sports performances and fall risk in older adults (1,2). The importance of balance control has been noted specifically in older adults to safely accomplish any types of motor tasks involving displacement of the entire body or even a few body segments in daily life, such as standing from a chair or simple gait motions. For these reasons, balance testing and exercise should focus on balance control challenging the center of gravity over the base of support while being confronted by destabilizing forces (1). Postural balance improved by balance training, therefore, not only prevents falls and subsequent injuries (2), but also optimizes motor performance and reduces sports injuries in a variety of athletic disciplines (1,3).

In daily life, reactive balance control strategies are required in response to a variety of postural perturbations, such as slips, trips, or being pushed by someone on a street. Reactive balance, which can be defined as an ability to recover a stable postural balance in response to a mechanical perturbation, requires rapid generation of postural muscles in the trunk, lower, or upper

limbs to induce proper reactions, such as swaying around the ankle or hip joints, taking a reactive step, or reaching to grasp a handhold (4). Delayed and inappropriate generation of corrective forces from the postural muscles may result in falling and injuries (5). Reactive balance control is supported by visual, vestibular, or somatosensory feedback, and it can be improved by repeated exposure to perturbations not only in healthy young and older adults, but individuals with neurological disease, such as multiple sclerosis (6).

In various clinical settings, exercises using ball throwing and catching activities have been broadly performed as a rehabilitation or performance training to improve balance and gait ability and to reduce the risk of athletic injuries (7,8). Recently, several researchers have conducted balance training studies consisting of self-initiated or external postural perturbations to improve reactive balance, described as compensatory postural adjustments, for healthy young adults (9), older adults (10,11), and stroke patients (12). They utilized a ball throwing activity as a self-initiated (internal) postural perturbation and a ball catching activity as an external perturbation. The ball throwing and catching exercises improved both anticipatory and compensatory postural adjustments in the populations. However, the participants, specifically the older adults or those with neurological disease, in the studies were exposed to potential risks, such as losing balance and falling during the training and testing sessions conducted in a dry-land condition. Thus, they needed additional safety measures, such as wearing a harness.

Various forms of exercises in an aquatic environment have been suggested as an effective and safer alternative method to exercises on dry land for people with a number of clinical conditions (13,14). A growing interest in the effectiveness of aquatic exercise fundamentally attribute to several physical properties of the water (13). The buoyancy of the water provides us with a supportive force and a reduction in joint loading. Viscous force and hydrostatic pressure

enhance sensory and proprioceptive awareness, which may be beneficial to postural control system (15). In addition, aquatic environment slows movements and enhances participants' confidence level (i.e., decrease in fear of falling) by potentializing their ability to attempt movement outside their base of support (15). Given these advantages of the aquatic environment, aquatic exercises have been widely implemented in the early stages of rehabilitation or used as a paradigm of aquatic cross-training (15,16). In general, it is recommended to start balance exercises with simple tasks and then incorporate more challenging tasks demanding a superior integration of visual, vestibular, and somatosensory systems (1). In aquatic environment, however, more difficult tasks requiring a higher level of sensory motor integration are theoretically possible when compared to the same tasks performed on land. Thus, aquatic exercises are specifically advisable for those who cannot tolerate land-based exercises or strive for novel training effects.

According to dynamic systems theory, variability in the movement systems affects each individual's behavior and consequentially help us adjust to a variety of constraints (personal, task-related, or environmental) (17). The aquatic environment increases the variability in movement behavior and therefore requires our neuromuscular system to adapt to it. In turn, considering that less skilled performers or injured individuals generally display weak adaptability to the constraints (17), the aquatic environment may be a more effective at improving balance control in various circumstances versus a dry-land environment. Despite the abovementioned attributes and benefits of the aquatic environment, there is void in the literature regarding how effective aquatic training exercises can be transferred to different tasks in a dry-land context when compared to land training exercises. Hence, the purpose of this study is to compare measures of reactive balance control before and after a ball throwing and catching task on land and in chest deep water in older adults. Considering water resistance producing overload during movements and hydrostatic pressure

constantly facilitating sensory awareness and motor functions in the aquatic environment, we hypothesize that participants in an aquatic exercise group will present greater improvements during the reactive balance assessment compared to those in a land exercise group.

Methods

Participants

A total of 44 older adults will be recruited for the current project. A power analysis was conducted using G*Power software package (Version 3.1.9.7, Kiel University, Germany). Using a result of the previous study comparing the dynamic balance between aquatic and land exercise groups (18), an a-priori power calculation (α=.05, and 1-β=.80) determined 38 participants will be needed for the intergroup comparison (i.e., aquatic vs. land exercise). Considering an approximately 15% dropout rate, a total of 44 subjects were expected to enter the study. Participants will be recruited from university and community settings via written flyers or word-of-mouth referrals. A pre-screening questionnaire will be utilized to estimate the eligibility of each participant.

Inclusion criteria: 1) age: 65 years or above; 2) have the ability to stand using a double-leg stance for one minute of time, 3) walk independently, 4) have normal or corrected to normal vision, 5) normal or corrected to normal hearing based on a qualitative assessment; and 6) fluent in English

Exclusion criteria: 1) any neurological or musculoskeletal disorder that inhibits the participation in the testing protocols; 2) a concussion in the 12 weeks before the study; 3) any cognitive deficiencies (e.g., memory, concentration, or attention disorder); 4) one or more 'yes' answered on the Physical Activity Readiness Questionnaire (PAR-Q); or 5) fear of water.

Intervention

The AE will be executed in a climate-controlled aquatic research laboratory, and the LE will be performed in a motion analysis laboratory. During AE, participants will stand barefoot on an adjustable floor of an aquatic treadmill (HydroWorx 2000TM, Middletown, PA), and the water depth will be adjusted to each participant's xiphoid process level. Participants will sit on a chair for five minutes before AE for environmental acclimatization.

Participants will engage in a single session of training consisting of 120 repetitions of a ball throwing and catching task using a volleyball (Wilson Sporting Goods Co., Chicago, IL, USA). This single-session approach was selected based on a previous study, reporting the long-lasting effects of a single session of intense reactive balance training (19). The participants will be asked to throw the ball at a self-paced high speed at the chest-to-shoulder level using two hands towards the trainer's hands positioned a 3-meter distance from them and 20-degree above an imaginary horizontal line at each participant's eye level. For the consistent height and trajectory of the ball across all training sessions, the trainer will kneel on the floor in front of the pool during AE sessions and stand on a step box during LE sessions. Then, they will catch the ball thrown at a moderate speed by the trainer directly toward the midline of their body, to the left or right of the midline, or to the closer or farther point from the trainer to induce fixed-support reactions and change-in-support reactions in both frontal and sagittal planes of the participants. The directions of the ball will be random and unpredictable, and the number of the directions will be proportionately included. After each repetition involving one throw and one catch, participants will be asked to return to the start position for the next repetition. The training session will be composed of 3 sets of 40 repetitions with a 2-min break after the first and second set. Of the 120 repetitions, the catches will follow 60 direct throws and 60 deceptive throws from the trainer to introduce the concepts of

response inhibition and action selection. For example, a deceptive throw will involve a deceptive action that provides misleading information about the direction of the ball projection (e.g., a throwing motion to the right without an actual throw) before the actual throw (e.g., throwing the ball to the left, which is the predetermined direction). While catching the ball, the participants will be allowed to select whichever strategy they wish and freely move their bodies to properly react and catch the ball. The Borg Scale for Rating of Perceived Exertion (RPE) will be used during each training set, and the heart rate will be recorded using a finger pulse oximeter (SportStat; Nonin Medical, Inc., Minneapolis, MN. USA) before and after each set to subjectively and objectively monitor the intensity of the training. Additional rest will be given if needed. The training procedures used previously by several trials have been reproduced and modified to generate reliable results in accordance with our research purpose (10,12,20,21). After each training session, subjective measures of satisfaction, difficulty, and safety levels will be additionally performed using a visual analogue scale (VAS, range: 1-10).

Outcome measurement

A modified lean-and-release technique devised by Bolton and Mansour (2020) will be implemented on land to assess reactive balance immediately before (pre), after (post), and one week after (follow-up) the single-session training (22). Lean-and-release techniques are commonly used to assess reactive balance in various older adult populations (23–25). This modified lean-and-release technique additionally introduces cognitive factors (i.e., response inhibition and action selection), which are important in certain situations. For safety purposes, participants will wear a harness, which is secured to the ceiling to prevent them from falling in case that they fail to recover balance. The participants will then stand with both feet hip-width apart on two separate, parallel

force plates (type 9260AA, Kistler AG, Winterthur, Switzerland). A horizontal cable will be attached to the safety harness at the midthoracic level and the other end to the wall behind the participants by a magnet. To simulate a forward fall (trip) situation, the experimenter will instruct participants to lean forward into a support cable while keeping both feet in contact with the floor and to remain relaxed (Fig 2). This position will require dorsiflexion of the ankle, as the rest of the body remains aligned. The forward lean position for each participant, measured by the ankle joint angle, will be determined as the minimal lean angle where a change-of-support reaction (i.e., forward step) is necessary to recover balance upon cable release (26). Then, the magnet will be deactivated to suddenly release the cable. There are two possible settings: 1) the leg block is placed in front of both legs, and a safety handle is uncovered; or 2) the leg block is removed, and the safety handle is covered. The leg block and handle cover will be controlled via computer-triggered, servo motors. The testing session will be comprised of three blocks: 1) REACH (grasping a safety handle using their right hand while maintaining both feet fixed), 2) STEP (stepping forward using any leg), and 3) RANDOM (random variations of STEP and REACH). To control vision during the third block, participants will wear liquid crystal goggles (Translucent Technologies Inc. Toronto, ON, Canada), that will be closed at the beginning of each trial and opened 400 ms before the cable is released. An experimenter will make careful note of the stepping leg used on each trial, and the participants will be free to step with either leg during testing. The timing of foot-off of the stepping foot will be recorded using the two parallel force plates. A force-sensitive resistor (B&L Engineering, Santa Ana, CA, USA) placed on the top surface of the safety handle and one large force plate in front of the two parallel force plates will be used to detect hand contact with the safety handle during REACH and foot contact during STEP, respectively. To control the predictability of the forthcoming reactions during the third block, the settings will be randomly

changed between trials via the data collection program. After sufficient practice trials to familiarize the participants with the stepping and reaching actions, five trials each of the REACH and STEP will be completed, and ten actual trials of RANDOM, including five STEP and five REACH actions in random orders, will be completed. Outcome measurements at post and follow-up will include all randomized participants within the original groups regardless of any discontinuation or deviation from intervention protocols.

During the testing, the quality of the compensatory reactions will be scored using the Reactive Postural Control section of the mini-BESTest (27,28). The scores will be ranged from 0 to 2. The scoring criteria for the STEP task will be as follows: 0) no reactive step or falling; 1) more than one reactive step; and 2) one successful reactive step. For the REACH task, the criteria were modified as follows: 0) no grasp reaction or falling; 1) grasp reaction with one or more reactive steps; 2) successful grasp reaction without a reactive step. During the RANDOM block, the number of accurate responses will be recorded. The testing will be video-recorded and assessed by two experimenters, who are blinded to the allocation.

Data processing and analysis

Force data in relation to the cable release will be extracted for further analysis to define the reaction time. Foot-off and foot contact will be defined as the time (ms) following the cable release at which the force under the stepping foot becomes less than and greater than 1% of the body weight, respectively (28). Hand contact with the handle during REACH will be defined as the time following the cable release at which the force sensitive resistor detects a force.

For the RANDOM block, response accuracy, defined as the percentage of accurate responses, will be additionally calculated. To represent a composite measure of accuracy and speed

of response, rapid response accuracy will be calculated using the ratio of response accuracy to the reaction time (%/ms), similar to the approach recently developed in a device measuring hand reactions (46). Foot-off and hand contact data will be used for the calculation.

Statistical analysis

Dependent variables included in the statistical analyses will include: 1) hand contact time during REACH; 2) foot-off time during STEP; 3) foot contact time during STEP; 4) rapid response accuracy during RANDOM; 5) mini-BESTest score of REACH; 6) mini-BESTest score of STEP; and 7) mini-BESTest score of RADOM. There are two independent variables including the training environments (AE vs. LE) and time (pre vs. post vs. follow-up).

The baseline differences in demographic data and all dependent variables between AE and LE groups will be analyzed using independent samples t-tests for data with a normal distribution and using the non-parametric Mann-Whitney U-test for data without normal distribution. On confirming the assumptions of the normal distribution (Kolmogorov–Smirnov test) and homogeneity of variances (Levene's test), dependent variables will be compared using 2 (group: AE, LE) × 3 (time: pre, post, follow-up) repeated-measures (RA) analysis of variance (ANOVA) with group as a between-group factor and time as a within-group factor. If baseline differences of the dependent variables occur, baseline values will be included as covariates. If the assumptions are violated, Kruskal-Wallis one-way ANOVA will be used for nonparametric variables. In addition, post-hoc analyses will be conducted using Tukey post hoc test or Mann-Whitney U test. The meaningfulness of statistical differences will be reported using Cohen's d effect sizes (ES) and confidence intervals (95%) for the parametrical variables and probability of superiority for dependent samples (PSdep) for the nonparametric variables [41]. Alpha will be set a priori at .05

for all statistical analyses. Missing data will be handled using multiple imputation, and all statistical analyses taking an intention-to-treat approach will be conducted with SPSS version 25 (SPSS Inc., Chicago, IL, USA).

References

1. Hrysomallis C. Balance Ability and Athletic Performance. Sports Med. 2011 Mar 1;41(3):221–32.
2. Sherrington C, Fairhall N, Wallbank G, Tiedemann A, Michaleff ZA, Howard K, et al. Exercise for preventing falls in older people living in the community: an abridged Cochrane systematic review. Br J Sports Med. 2020 Aug 1;54(15):885–91.
3. Sañudo B, Sánchez-Hernández J, Bernardo-Filho M, Abdi E, Taiar R, Núñez J. Integrative Neuromuscular Training in Young Athletes, Injury Prevention, and Performance Optimization: A Systematic Review. Appl Sci. 2019 Jan;9(18):3839.
4. Shumway-Cook A, Woollacott MH. Motor Control: Translating Research Into Clinical Practice. Lippincott Williams & Wilkins; 2017. 634 p.
5. Shumway-Cook A, Woollacott MH. Part 2. Postural Control. In: Motor Control: Translating Research Into Clinical Practice. Wolters Kluwer; 2017.
6. Mohamed Suhaimy MSB, Okubo Y, Hoang PD, Lord SR. Reactive Balance Adaptability and Retention in People With Multiple Sclerosis: A Systematic Review and Meta-Analysis.

Neurorehabil Neural Repair. 2020 Aug 1;34(8):675–85.

7. Shin S-S, An D-H. The Effect of Motor Dual-task Balance Training on Balance and Gait of Elderly Women. J Phys Ther Sci. 2014;26(3):359–61.

8. Pasanen K, Parkkari J, Pasanen M, Hiilloskorpi H, Mäkinen T, Järvinen M, et al. Neuromuscular training and the risk of leg injuries in female floorball players: cluster randomised controlled study. BMJ [Internet]. 2008 Jul 1 [cited 2020 Oct 21];337. Available from: https://www.bmj.com/content/337/bmj.a295

9. Kanekar N, Aruin AS. Improvement of anticipatory postural adjustments for balance control: Effect of a single training session. J Electromyogr Kinesiol. 2015 Apr 1;25(2):400–5.

10. Aruin AS, Kanekar N, Lee Y-J, Ganesan M. Enhancement of anticipatory postural adjustments in older adults as a result of a single session of ball throwing exercise. Exp Brain Res. 2015 Feb 1;233(2):649–55.

11. Jagdhane S, Kanekar N, S. Aruin A. The Effect of a Four-Week Balance Training Program on Anticipatory Postural Adjustments in Older Adults: A Pilot Feasibility Study. Curr Aging Sci. 2016 Nov 1;9(4):295–300.

12. Curuk E, Lee Y, Aruin AS. Individuals with stroke improve anticipatory postural adjustments after a single session of targeted exercises. Hum Mov Sci. 2020 Feb 1;69:102559.

13. Aquatic Exercise Association. Aquatic Fitness Professional Manual. Human Kinetics; 2017. 420 p.

14. Kim Y, Vakula MN, Waller B, Bressel E. A systematic review and meta-analysis comparing the effect of aquatic and land exercise on dynamic balance in older adults. BMC Geriatr. 2020 Aug 25;20(1):302.

15. Geigle PR, Cheek W, Gould M, Hunt H, Shafiq B. Aquatic physical therapy for balance: the interaction of somatosensory and hydrodynamic principles. J Aquat Phys. 1997;5(1):4–10.

16. Haff GG. Aquatic Cross Training for Athletes: Part I. Strength Cond J. 2008 Apr;30(2):18–26.

17. Davids K, Glazier P, Araújo D, Bartlett R. Movement Systems as Dynamical Systems. Sports Med. 2003 Apr 1;33(4):245–60.

18. Douris P, Southard V, Varga C, Schauss W, Gennaro C, Reiss A. The effect of land and aquatic exercise on balance scores in older adults. J Geriatr Phys Ther. 2003 Mar;26(1):3–6.

19. Bhatt T, Pai Y-C. Prevention of Slip-Related Backward Balance Loss: The Effect of Session Intensity and Frequency on Long-Term Retention. Arch Phys Med Rehabil. 2009 Jan 1;90(1):34–42.

20. Lee Y, Goyal N, Luna G, Aruin AS. Role of a single session of ball throwing exercise on

postural control in older adults with mild cognitive impairment. Eur J Appl Physiol. 2020 Feb 1;120(2):443–51.

21. Aruin AS, Ganesan M, Lee Y. Improvement of postural control in individuals with multiple sclerosis after a single-session of ball throwing exercise. Mult Scler Relat Disord. 2017 Oct 1;17:224–9.

22. Bolton DAE, Mansour M. A Modified Lean and Release Technique to Emphasize Response Inhibition and Action Selection in Reactive Balance. JoVE J Vis Exp. 2020 Mar 19;(157):e60688.

23. Ochi A, Yokoyama S, Abe T, Yamada K, Tateuchi H, Ichihashi N. Differences in muscle activation patterns during step recovery in elderly women with and without a history of falls. Aging Clin Exp Res. 2014 Apr 1;26(2):213–20.

24. Inness EL, Mansfield A, Biasin L, Brunton K, Bayley M, McIlroy WE. Clinical implementation of a reactive balance control assessment in a sub-acute stroke patient population using a 'lean-and-release' methodology. Gait Posture. 2015 Feb 1;41(2):529–34.

25. Mansfield A, Inness EL, Komar J, Biasin L, Brunton K, Lakhani B, et al. Training Rapid Stepping Responses in an Individual With Stroke. Phys Ther. 2011 Jun 1;91(6):958–69.

26. Bolton DAE, Cole DM, Butler B, Mansour M, Rydalch G, McDannald DW, et al. Motor preparation for compensatory reach-to-grasp responses when viewing a wall-mounted safety handle. Cortex J Devoted Study Nerv Syst Behav. 2019 Aug;117:135–46.

27. Franchignoni F, Horak F, Godi M, Nardone A, Giordano A. Using psychometric techniques to improve the Balance Evaluation Systems Test: the mini-BESTest. J Rehabil Med. 2010 Apr 1;42(4):323–31.

28. Borrelli JR, Junod CA, Inness EL, Jones S, Mansfield A, Maki BE. Clinical assessment of reactive balance control in acquired brain injury: A comparison of manual and cable release-from-lean assessment methods. Physiother Res Int. 2019;24(4):e1787.
